# Supplementary material for: AlphaFold2-Based Characterization of Apo and Holo Protein Structures and Conformational Ensembles Using Randomized Alanine Sequence Scanning Adaptation: Capturing Shared Signature Dynamics and Ligand-Induced Conformational Changes
Source: Int J Mol Sci. 2024 Dec 2;25(23):12968. doi: 10.3390/ijms252312968 (PMC11641424; doi:10.3390/ijms252312968)
Supplement: Supplementary file 1 [file ijms-25-12968-s001.zip › SUPPLEMENTARY_MATERIALS/SUPPLEMENTARY_MATERIALS_IJMS_REVISION.pdf]

Supplementary Materials

AlphaFold2-Based Characterization of Apo and Holo Protein Structures and Conformational Ensembles Using Randomized Alanine Sequence Scanning Adaptation: Capturing Shared Signature Dynamics and Ligand-Induced Conformational Changes

Nishank Raisinghani, [1] Vedant Parikh,[1] Brandon Foley,[1] Gennady Verkhivker[1,2*]

Keck Center for Science and Engineering, Graduate Program in Computational and Data Sciences, Schmid College of Science and Technology, Chapman University, Orange, CA 92866, United States of America

[2] Department of Biomedical and Pharmaceutical Sciences, Chapman University School of Pharmacy, Irvine, CA 92618, United States of America

* Correspondence: verkhivk@chapman.edu; Tel.: +1-714-516-4586 (G.V)

Received: date; Accepted: date; Published: date

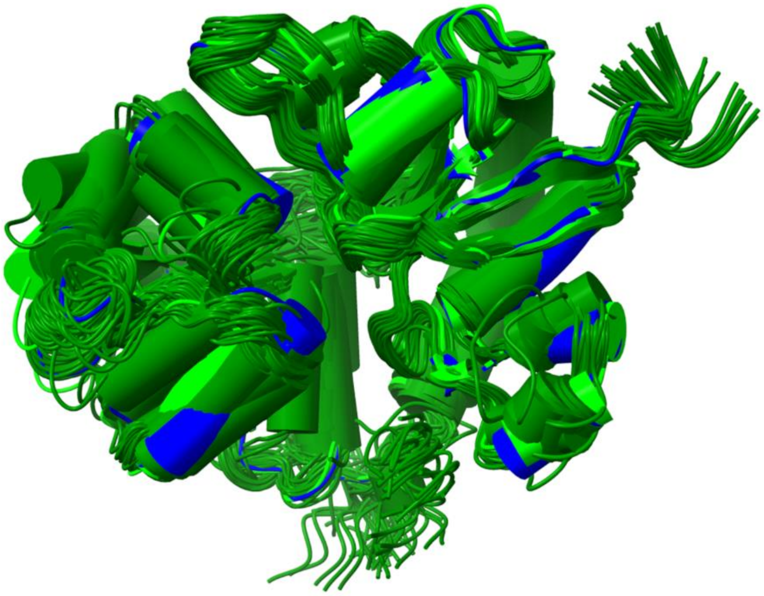

Figure S1. The AF2-RASS predicted conformational ensembles for apo-holo proteins. The apo-holo structure of GluR2 ligand binding core (apo PDB :1fto, holo PDB: 1ftm). The AF2-RASS generated conformations are shown in green ribbons, the crystallographic apo forms are in green ribbons and the crystallographic holo forms are in blue ribbons.

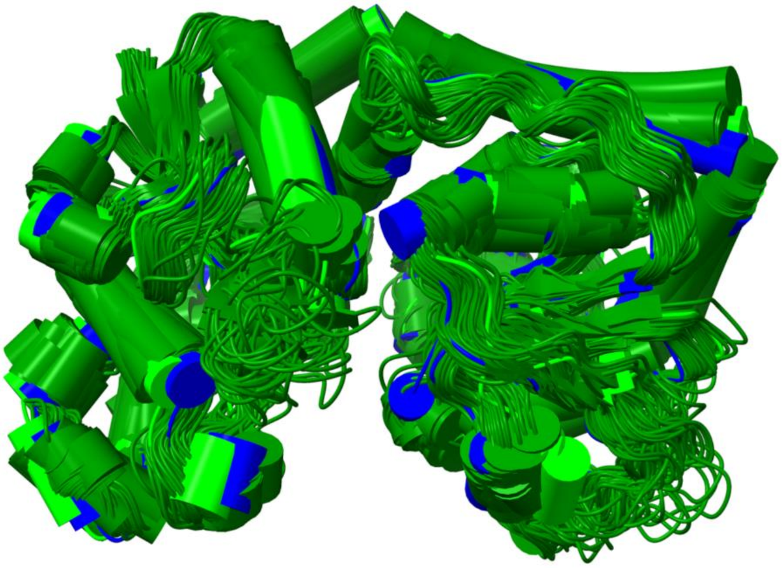

Figure S2. The AF2-RASS predicted conformational ensembles for apo-holo proteins. DNA Beta-Glucosyl-transferase (apo PDB :1jej, holo PDB: 1jg6). The AF2-RASS generated conformations are shown in green ribbons, the crystallographic apo forms are in green ribbons and the crystallographic holo forms are in blue ribbons.

The AF2-RASS predicted conformational ensembles for apo-holo proteins (A) The apo-holo structure of GluR2 ligand binding core (apo PDB :1fto, holo PDB: 1ftm). (B) DNA Beta-Glucosyl-transferase (apo PDB :1jej, holo PDB: 1jg6). (C) D-Allose binding protein (apo PDB :1gud, holo PDB: 1rpj). (D) D-Ribose binding protein (apo PDB: 1urp; holo PDB :2dri). (E) 5-Enolpyruvylshikimate-3-phosphate synthase (apo PDB: 1rf5; holo PDB :1rf4). (F) Osmo-protection protein (apo PDB: 1sw5; holo PDB :1sw4). (G) Guanylate kinase (apo PDB: 1ex6; holo PDB :1ex7). (H) Hexokinase (apo PDB: 2e2n; holo PDB :2e2o). (I) ABC transporter OpuC (apo PDB: 3ppn; holo PDB :3ppr). (J) T4 Lysozyme L99A (apo PDB: 4w51; holo PDB :4w58). (K) Human cellular retinol binding protein 1 (apo PDB: 5h9a; holo PDB :6e5l). (L) Lipoprotein LpqN (apo PDB: 6epd; holo PDB :6e5f).

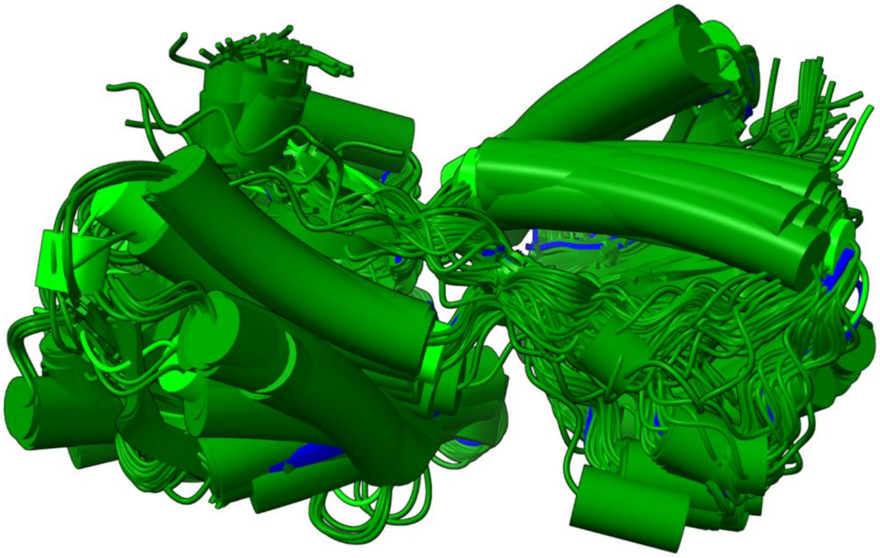

Figure S3. The AF2-RASS predicted conformational ensembles for apo-holo proteins. D-Allose binding protein (apo PDB :1gud, holo PDB: 1rpj). The AF2-RASS generated conformations are shown in green ribbons, the crystallographic apo forms are in green ribbons and the crystallographic holo forms are in blue ribbons.

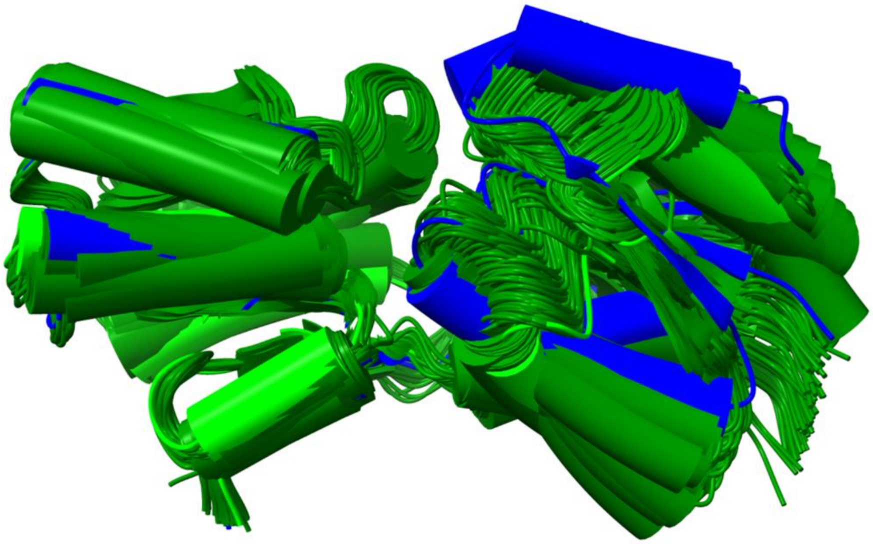

Figure S4. The AF2-RASS predicted conformational ensembles for apo-holo proteins. D-Ribose binding protein (apo PDB: 1urp; holo PDB :2dri). The AF2-RASS generated conformations are shown in green ribbons, the crystallographic apo forms are in green ribbons and the crystallographic holo forms are in blue ribbons.

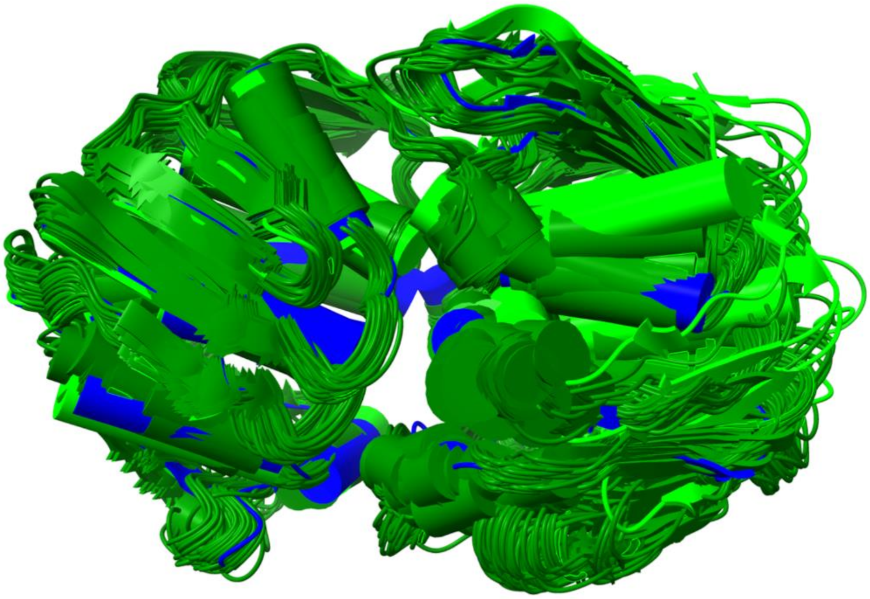

Figure S5. The AF2-RASS predicted conformational ensembles for apo-holo proteins. 5-Enolpyruvylshikimate-3-phosphate synthase (apo PDB: 1rf5; holo PDB :1rf4). The AF2-RASS generated conformations are shown in green ribbons, the crystallographic apo forms are in green ribbons and the crystallographic holo forms are in blue ribbons.

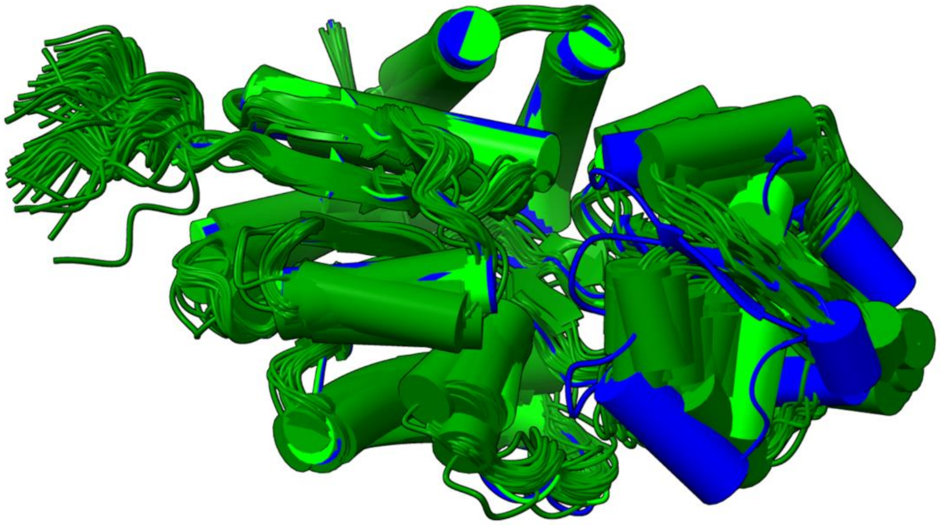

Figure S6. The AF2-RASS predicted conformational ensembles for apo-holo proteins. Osmo-protection protein (apo PDB: 1sw5; holo PDB :1sw2). The AF2-RASS generated conformations are shown in green ribbons, the crystallographic apo forms are in green ribbons and the crystallographic holo forms are in blue ribbons.

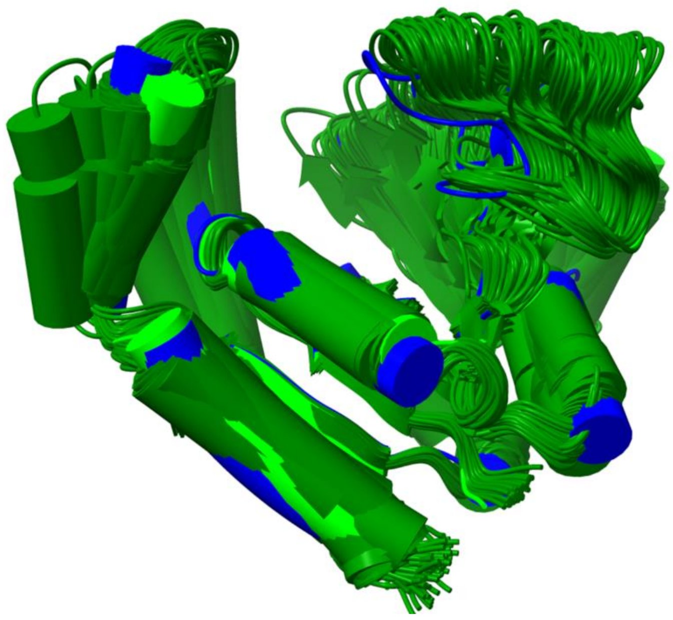

Figure S7. The AF2-RASS predicted conformational ensembles for apo-holo proteins. Guanylate kinase (apo PDB: 1ex6; holo PDB :1ex7). The AF2-RASS generated conformations are shown in green ribbons, the crystallographic apo forms are in green ribbons and the crystallographic holo forms are in blue ribbons.

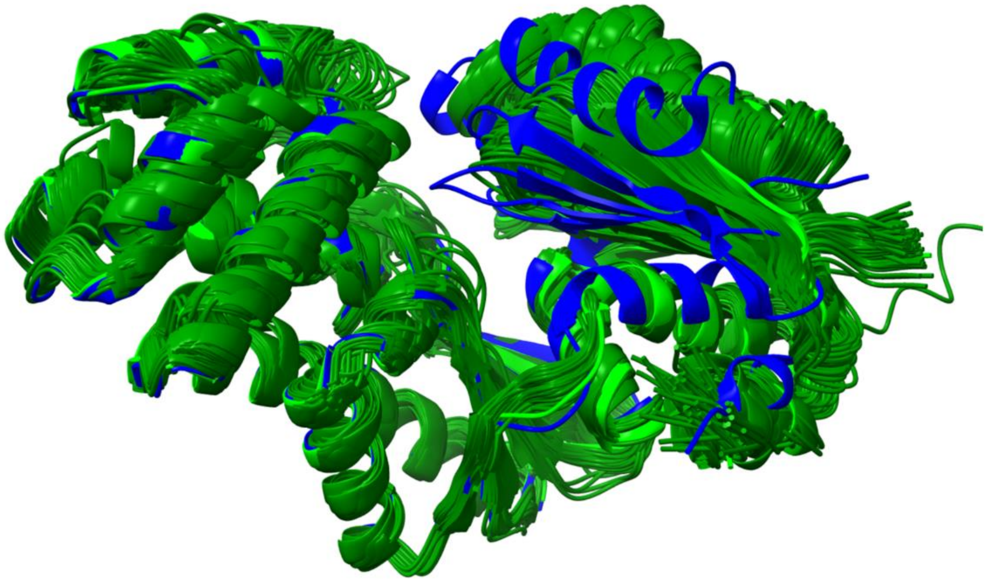

Figure S8. The AF2-RASS predicted conformational ensembles for apo-holo proteins. Hexokinase (apo PDB: 2e2n; holo PDB :2e2o). The AF2-RASS generated conformations are shown in green ribbons, the crystallographic apo forms are in green ribbons and the crystallographic holo forms are in blue ribbons.

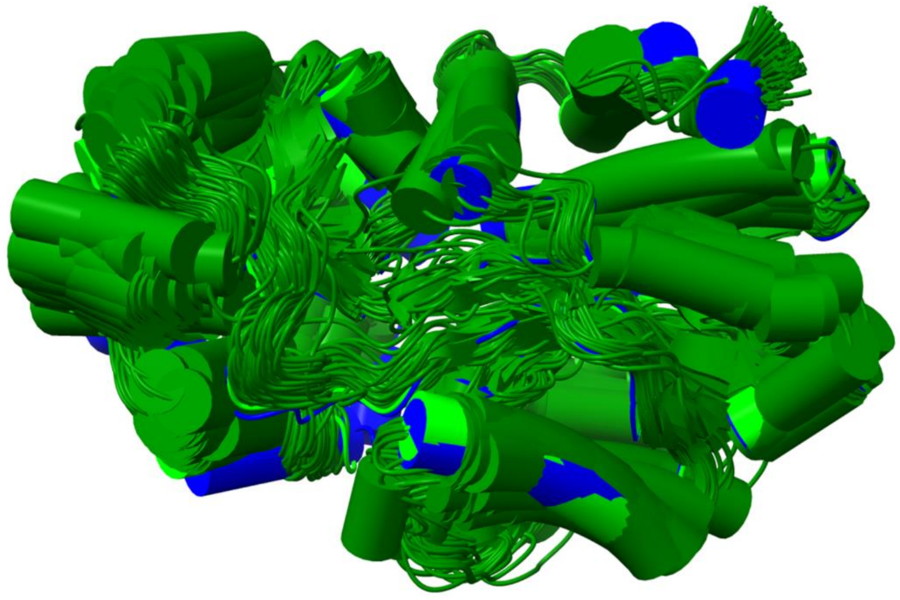

Figure S9. The AF2-RASS predicted conformational ensembles for apo-holo proteins. ABC transporter OpuC (apo PDB: 3ppn; holo PDB :3ppr). The AF2-RASS generated conformations are shown in green ribbons, the crystallographic apo forms are in green ribbons and the crystallographic holo forms are in blue ribbons.

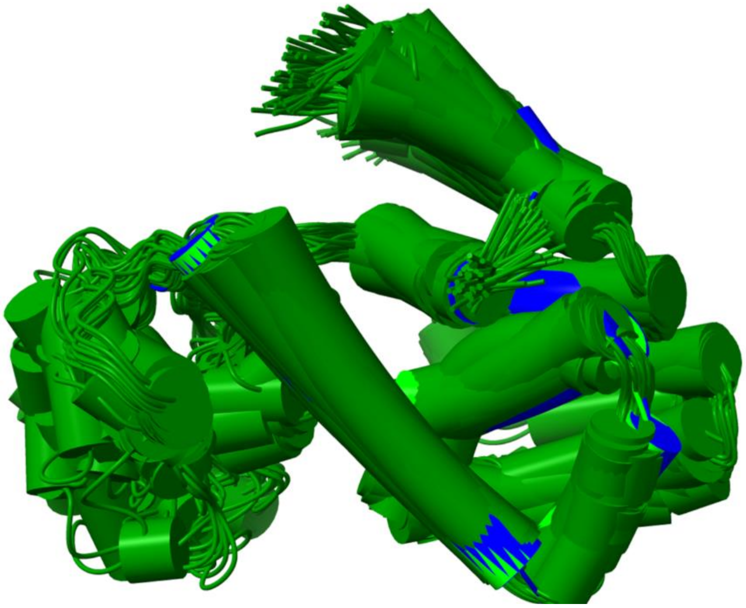

Figure S10. The AF2-RASS predicted conformational ensembles for apo-holo proteins. T4 Lysozyme L99A (apo PDB: 4w51; holo PDB :4w58). The AF2-RASS generated conformations are shown in green ribbons, the crystallographic apo forms are in green ribbons and the crystallographic holo forms are in blue ribbons.

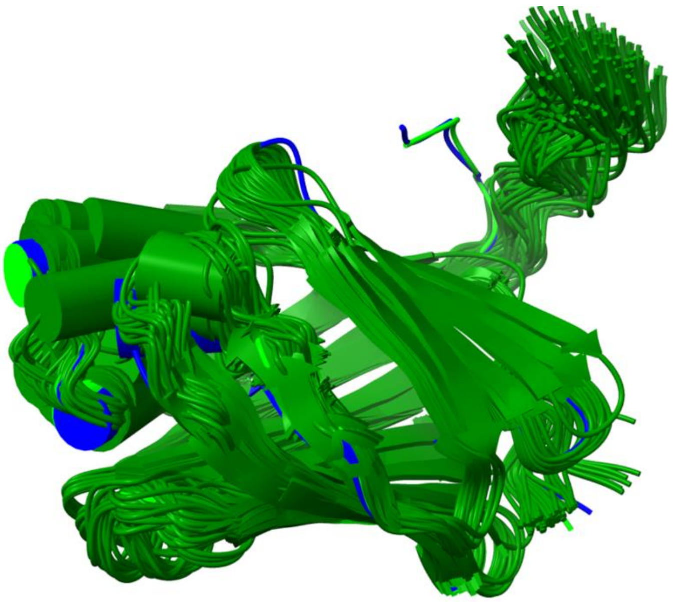

Figure S11. The AF2-RASS predicted conformational ensembles for apo-holo proteins. Human cellular retinol binding protein 1 (apo PDB: 5h9a; holo PDB :6e5l). The AF2-RASS generated conformations are shown in green ribbons, the crystallographic apo forms are in green ribbons and the crystallographic holo forms are in blue ribbons.

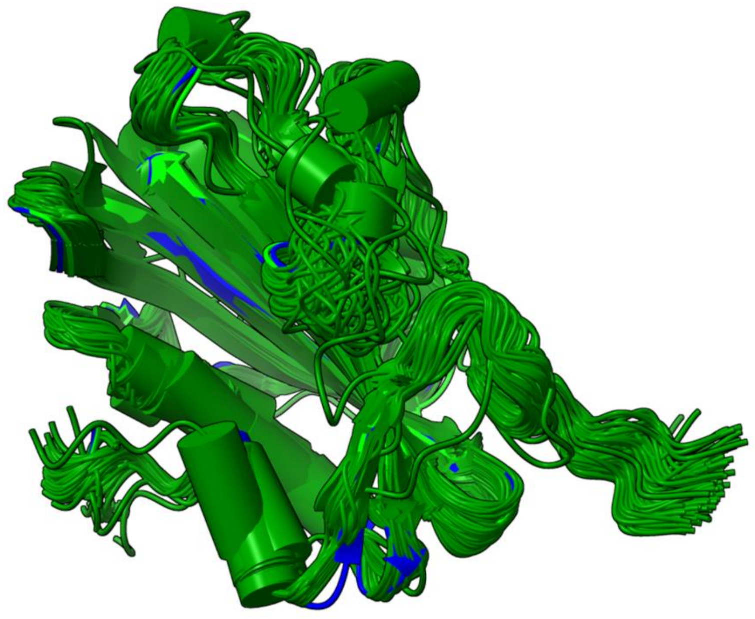

Figure S12. The AF2-RASS predicted conformational ensembles for apo-holo proteins. Lipoprotein LpqN (apo PDB: 6epd; holo PDB :6e5f). The AF2-RASS generated conformations are shown in green ribbons, the crystallographic apo forms are in green ribbons and the crystallographic holo forms are in blue ribbons.
