# Supplementary figures and images for: AlphaFold2-Based Characterization of Apo and Holo Protein Structures and Conformational Ensembles Using Randomized Alanine Sequence Scanning Adaptation: Capturing Shared Signature Dynamics and Ligand-Induced Conformational Changes
Source: Int J Mol Sci. 2024 Dec 2;25(23):12968. doi: 10.3390/ijms252312968 (PMC11641424; doi:10.3390/ijms252312968)

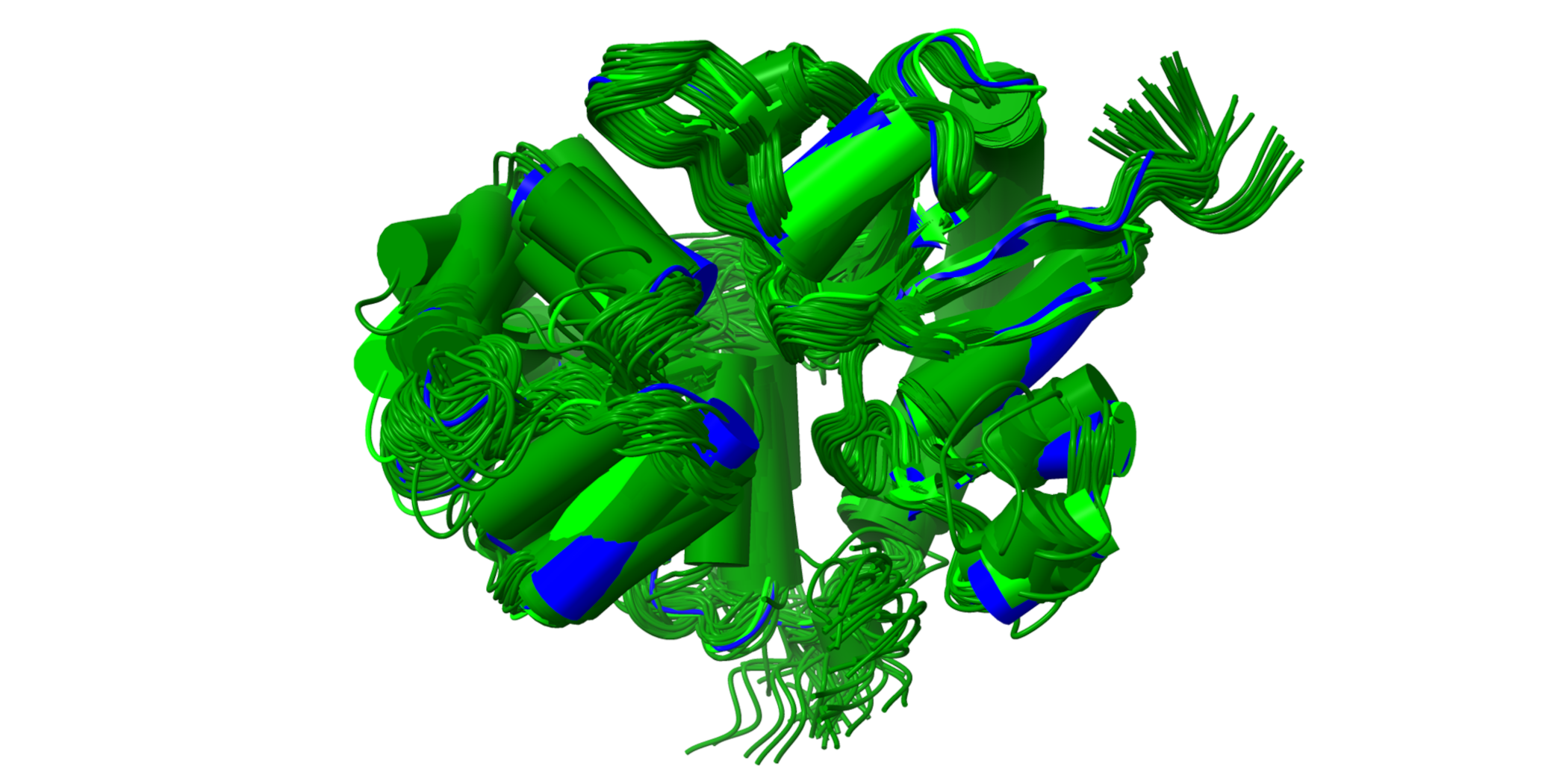

Supplement: Supplementary file 1 [file ijms-25-12968-s001.zip › SUPPLEMENTARY_MATERIALS/FigureS1.tif]

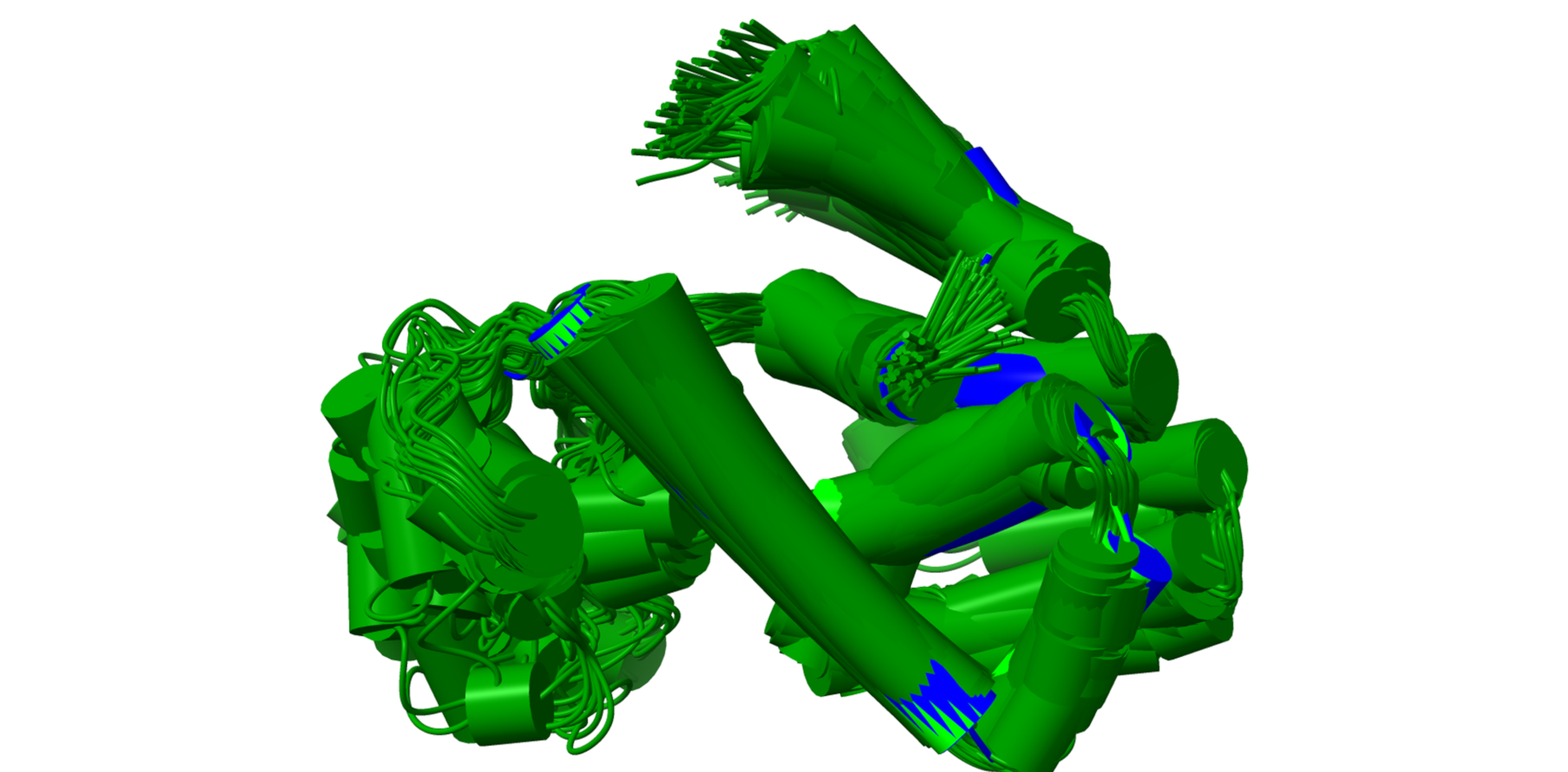

Supplement: Supplementary file 1 [file ijms-25-12968-s001.zip › SUPPLEMENTARY_MATERIALS/FigureS10.tif]

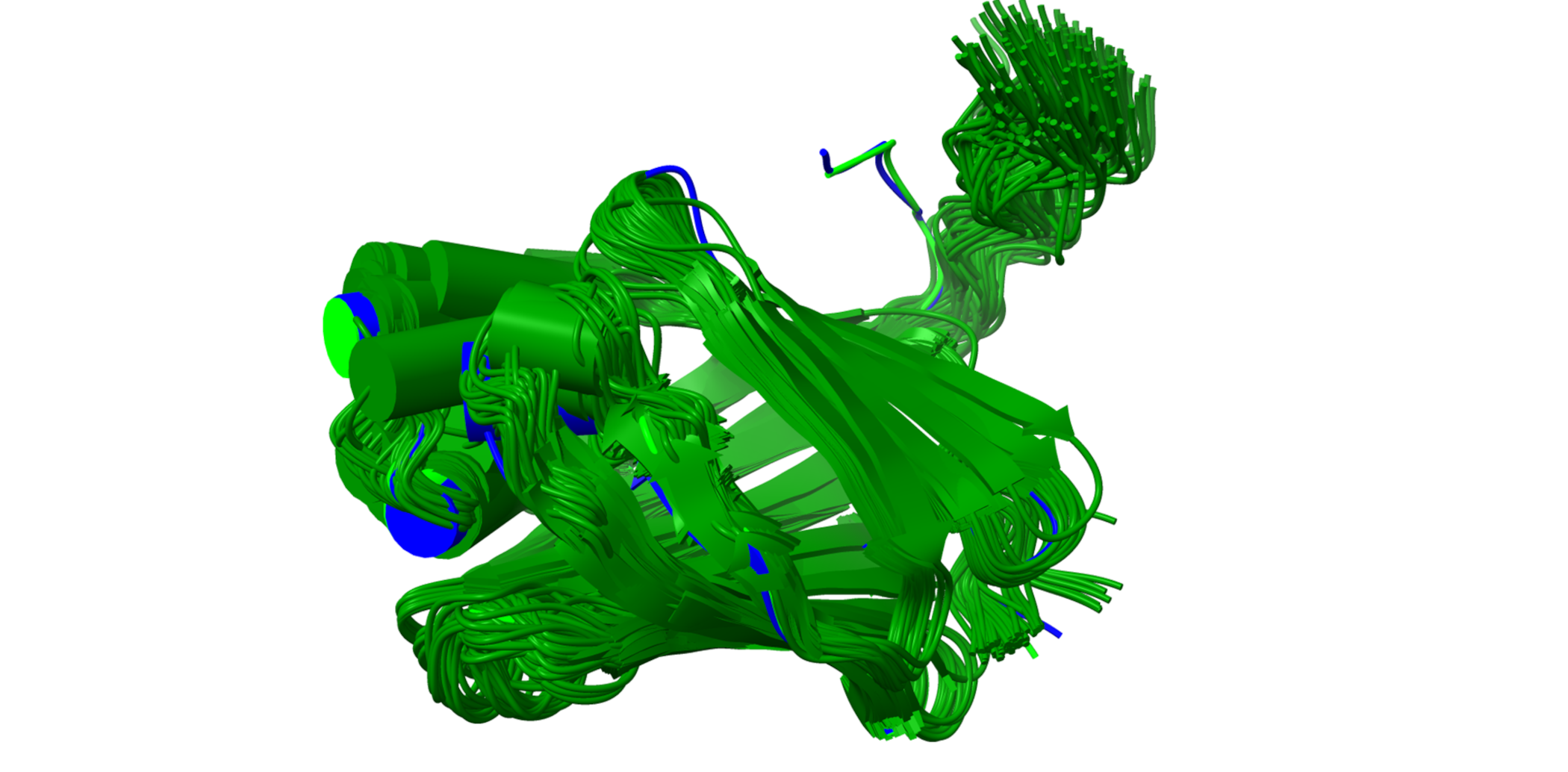

Supplement: Supplementary file 1 [file ijms-25-12968-s001.zip › SUPPLEMENTARY_MATERIALS/FigureS11.tif]

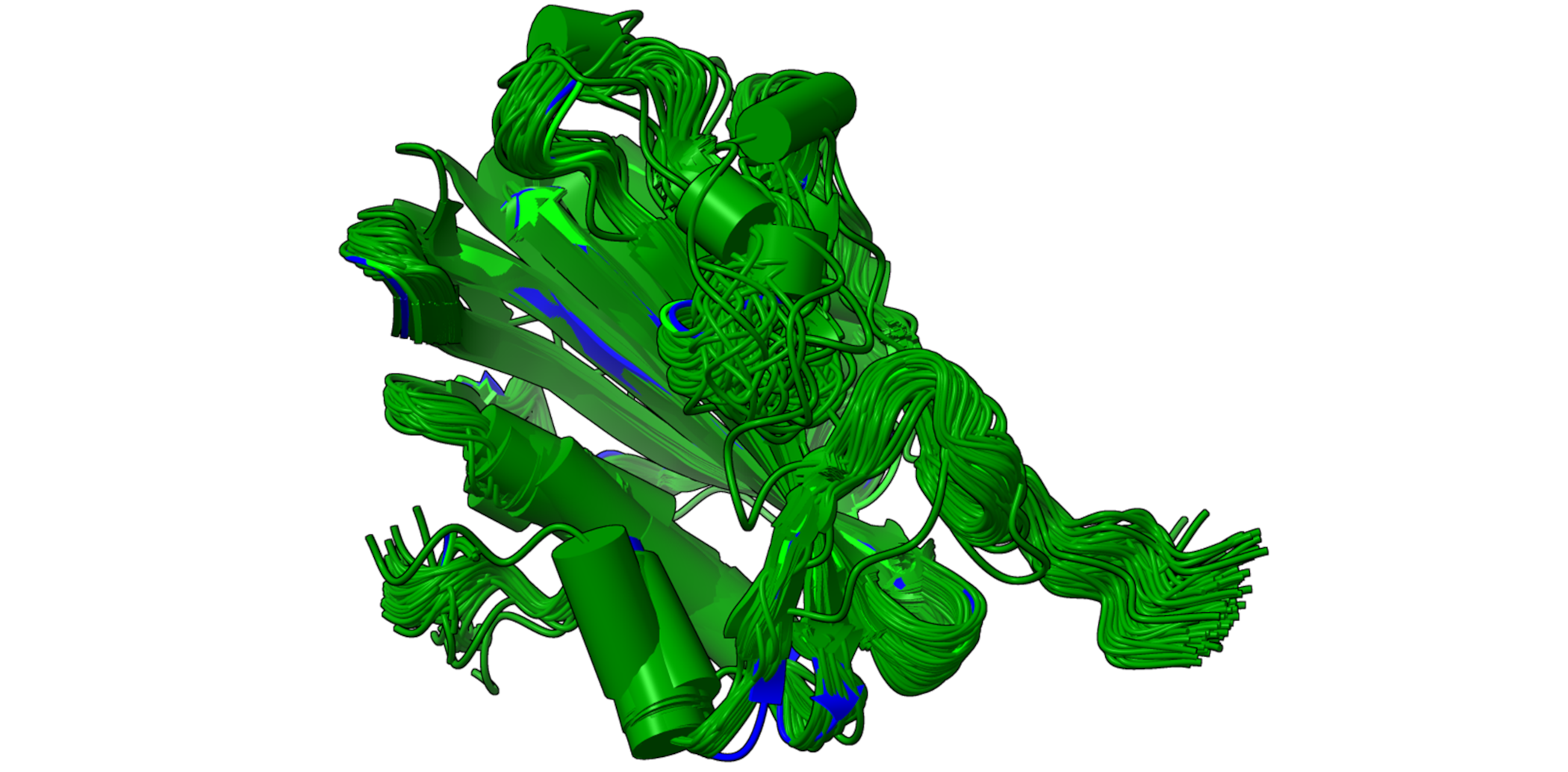

Supplement: Supplementary file 1 [file ijms-25-12968-s001.zip › SUPPLEMENTARY_MATERIALS/FigureS12.tif]

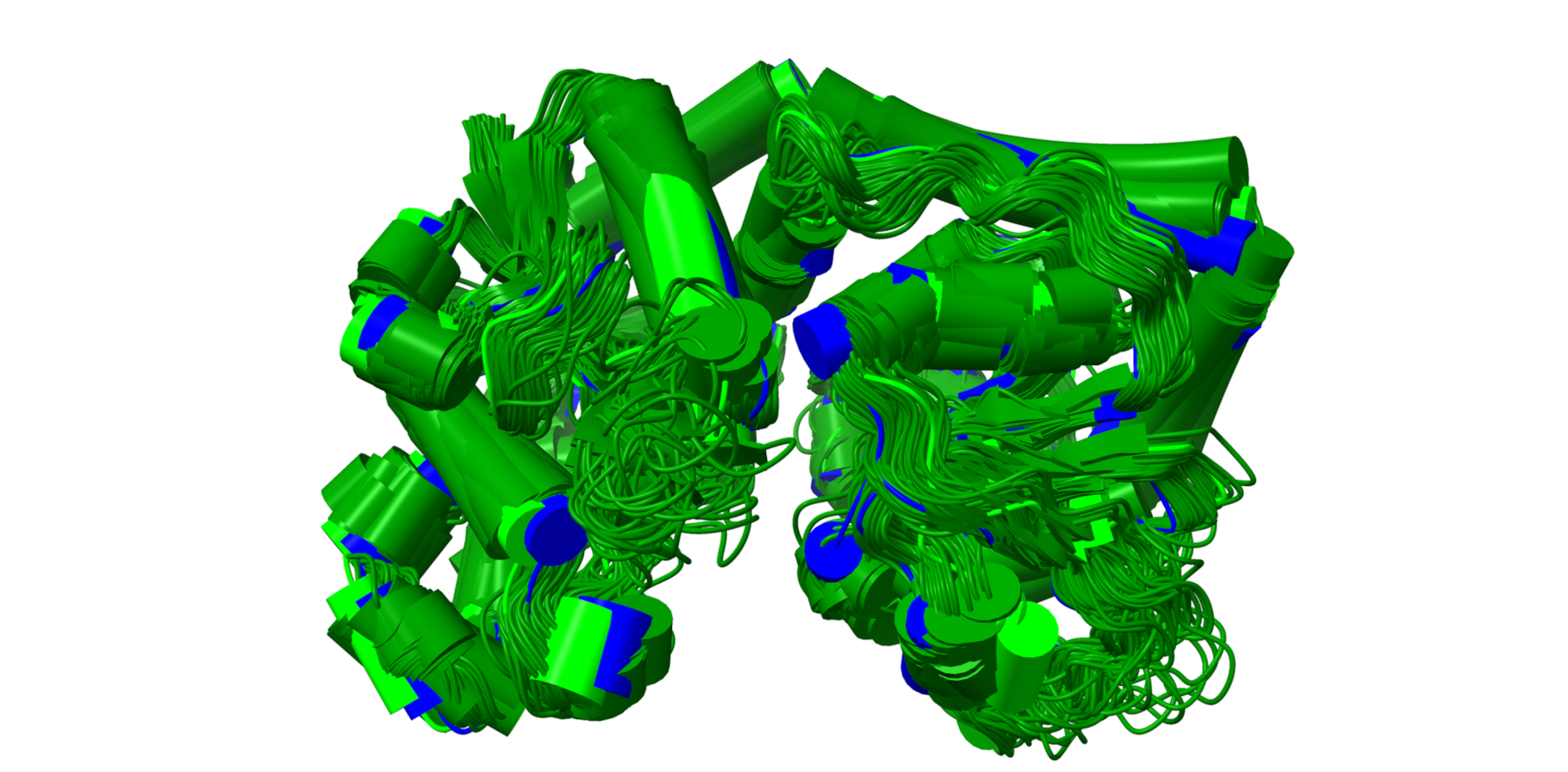

Supplement: Supplementary file 1 [file ijms-25-12968-s001.zip › SUPPLEMENTARY_MATERIALS/FigureS2.tif]

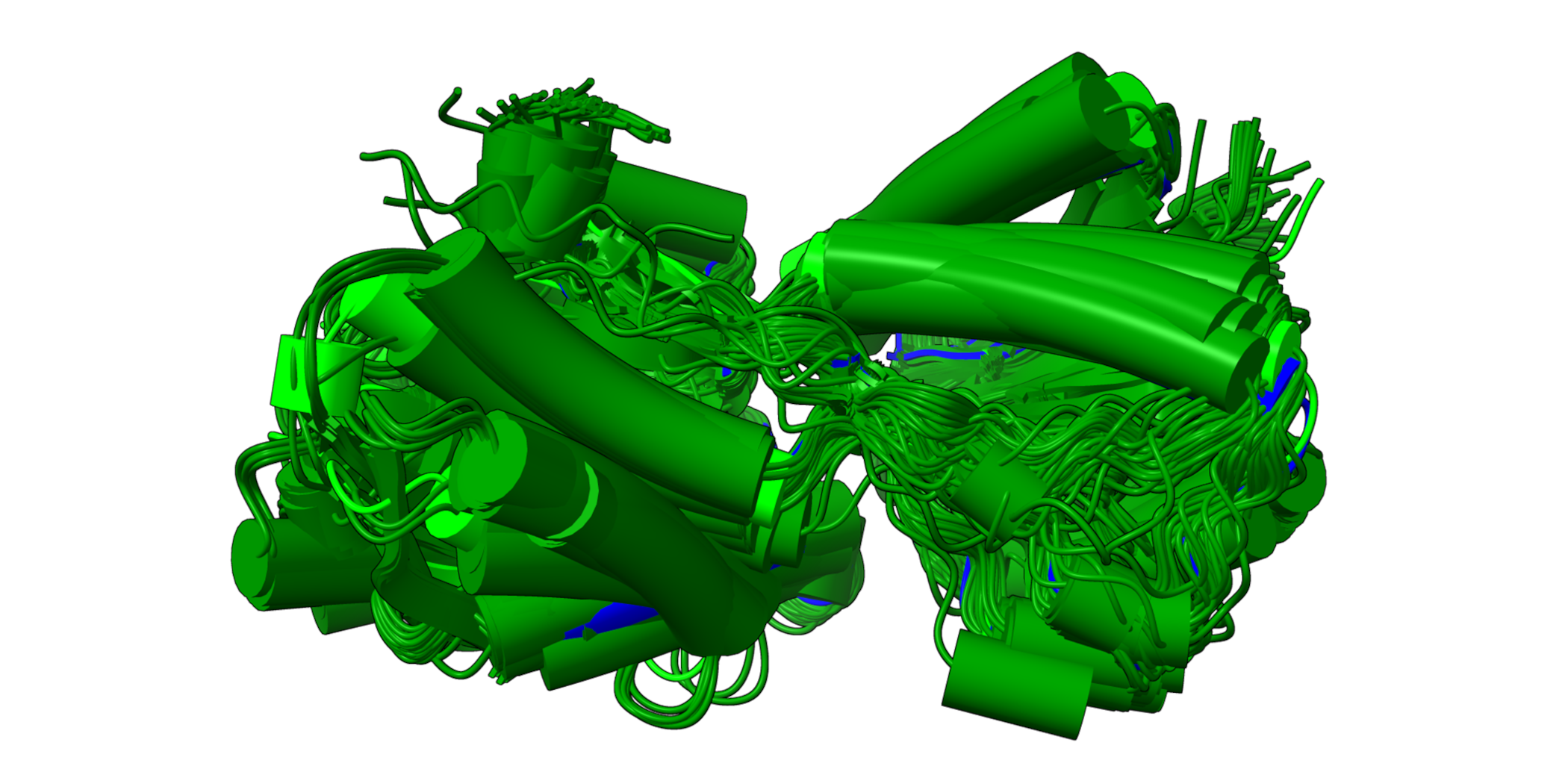

Supplement: Supplementary file 1 [file ijms-25-12968-s001.zip › SUPPLEMENTARY_MATERIALS/FigureS3.tif]

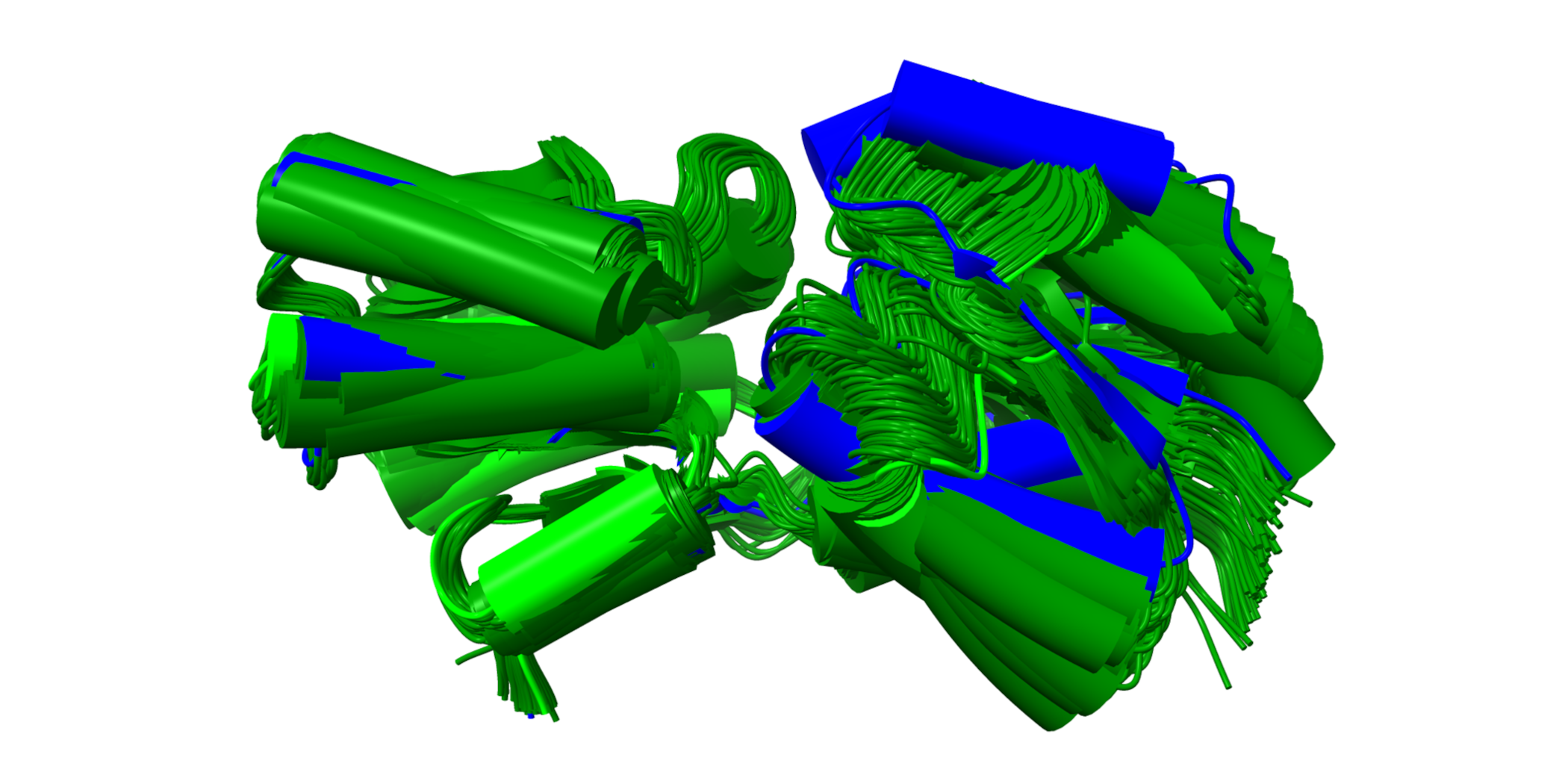

Supplement: Supplementary file 1 [file ijms-25-12968-s001.zip › SUPPLEMENTARY_MATERIALS/FigureS4.tif]

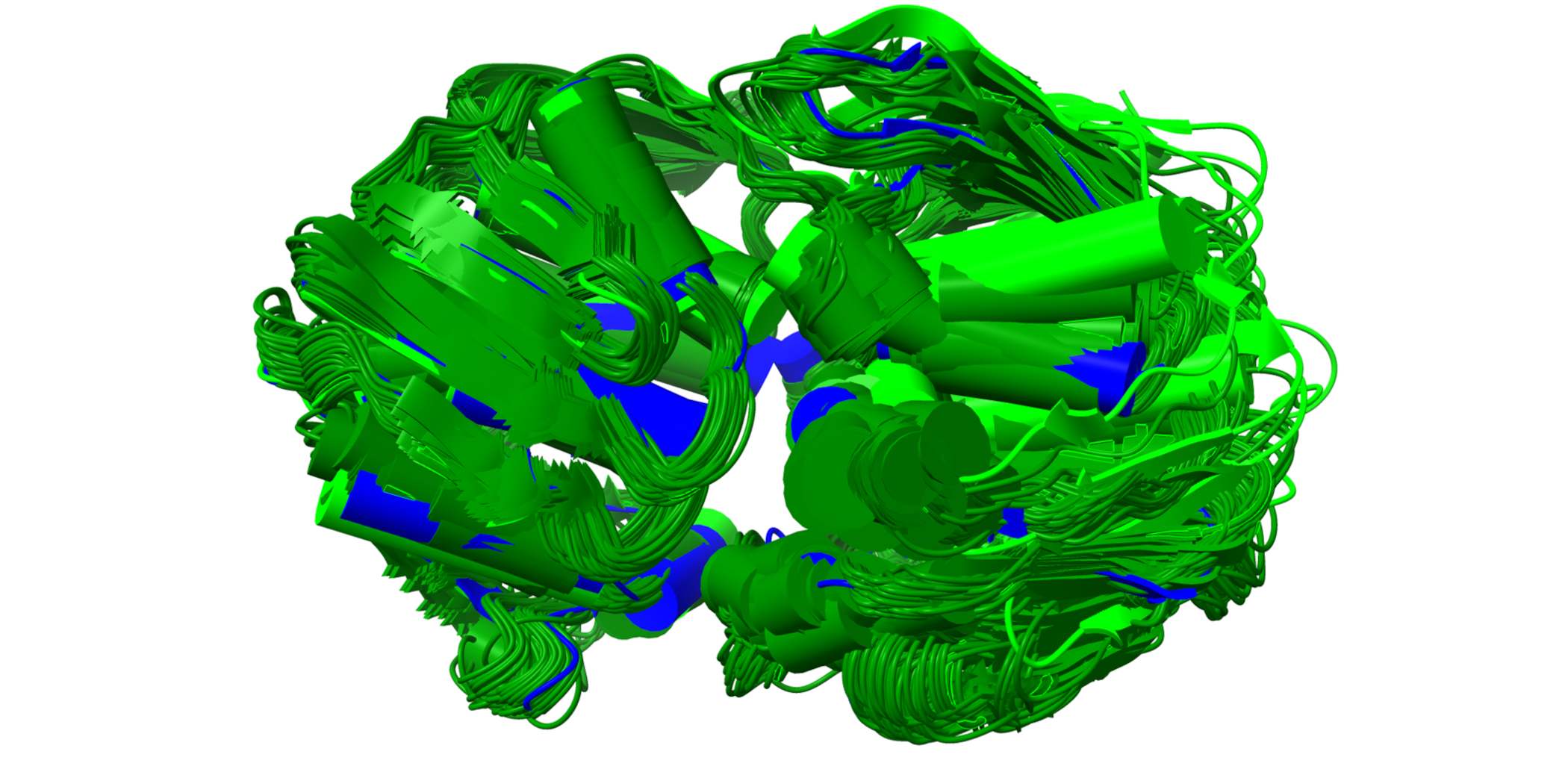

Supplement: Supplementary file 1 [file ijms-25-12968-s001.zip › SUPPLEMENTARY_MATERIALS/FigureS5.tif]

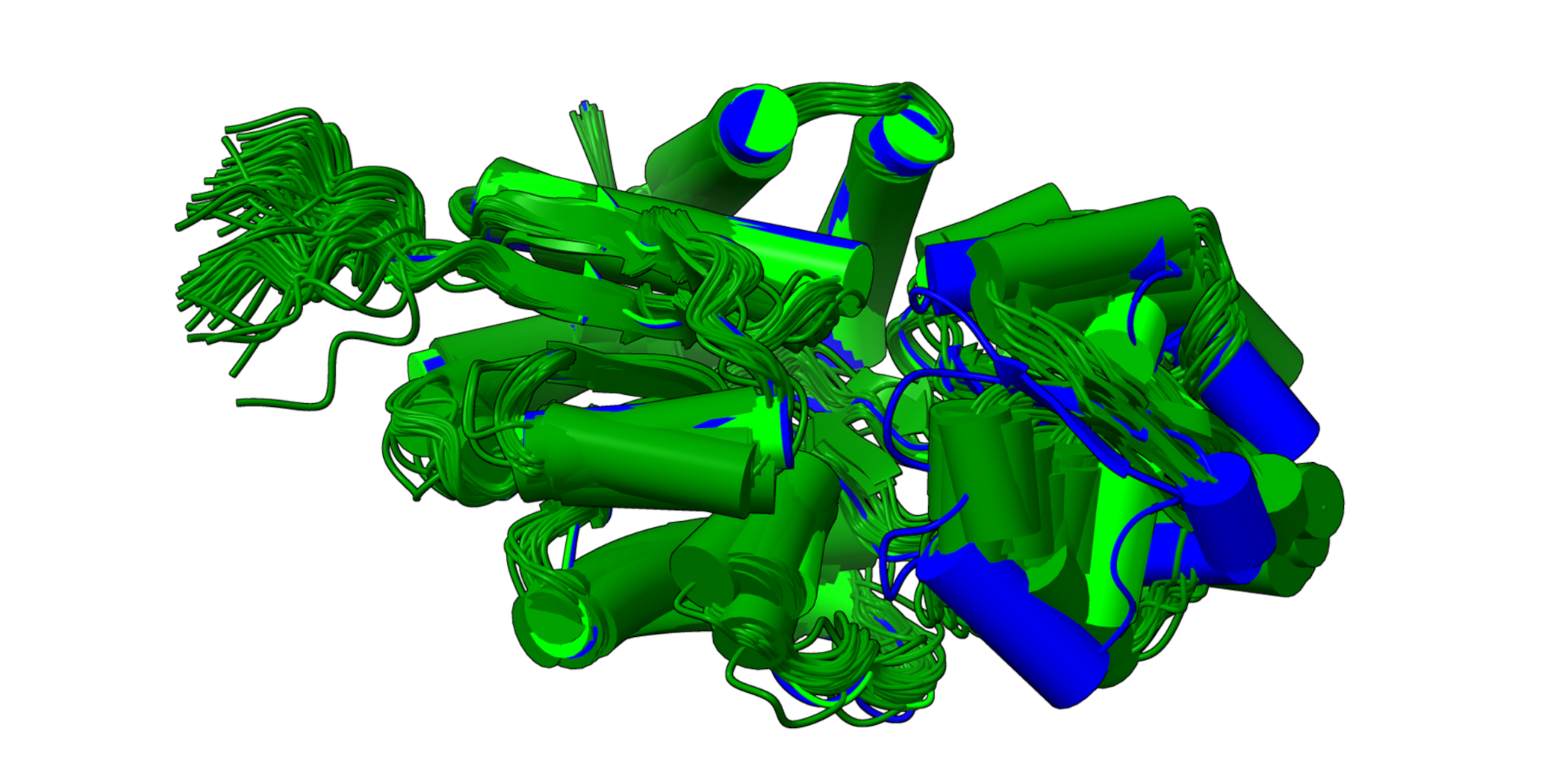

Supplement: Supplementary file 1 [file ijms-25-12968-s001.zip › SUPPLEMENTARY_MATERIALS/FigureS6.tif]

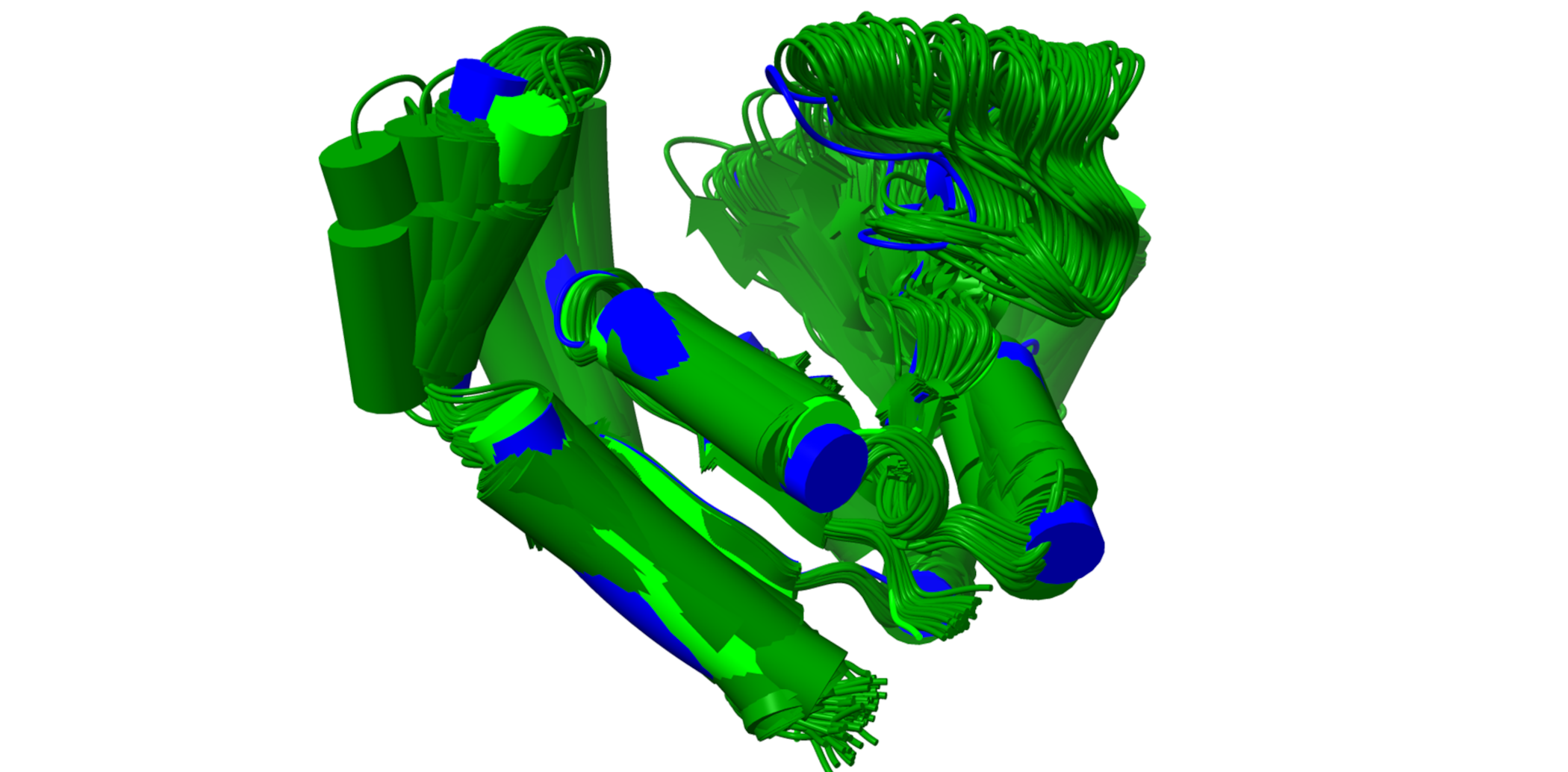

Supplement: Supplementary file 1 [file ijms-25-12968-s001.zip › SUPPLEMENTARY_MATERIALS/FigureS7.tif]

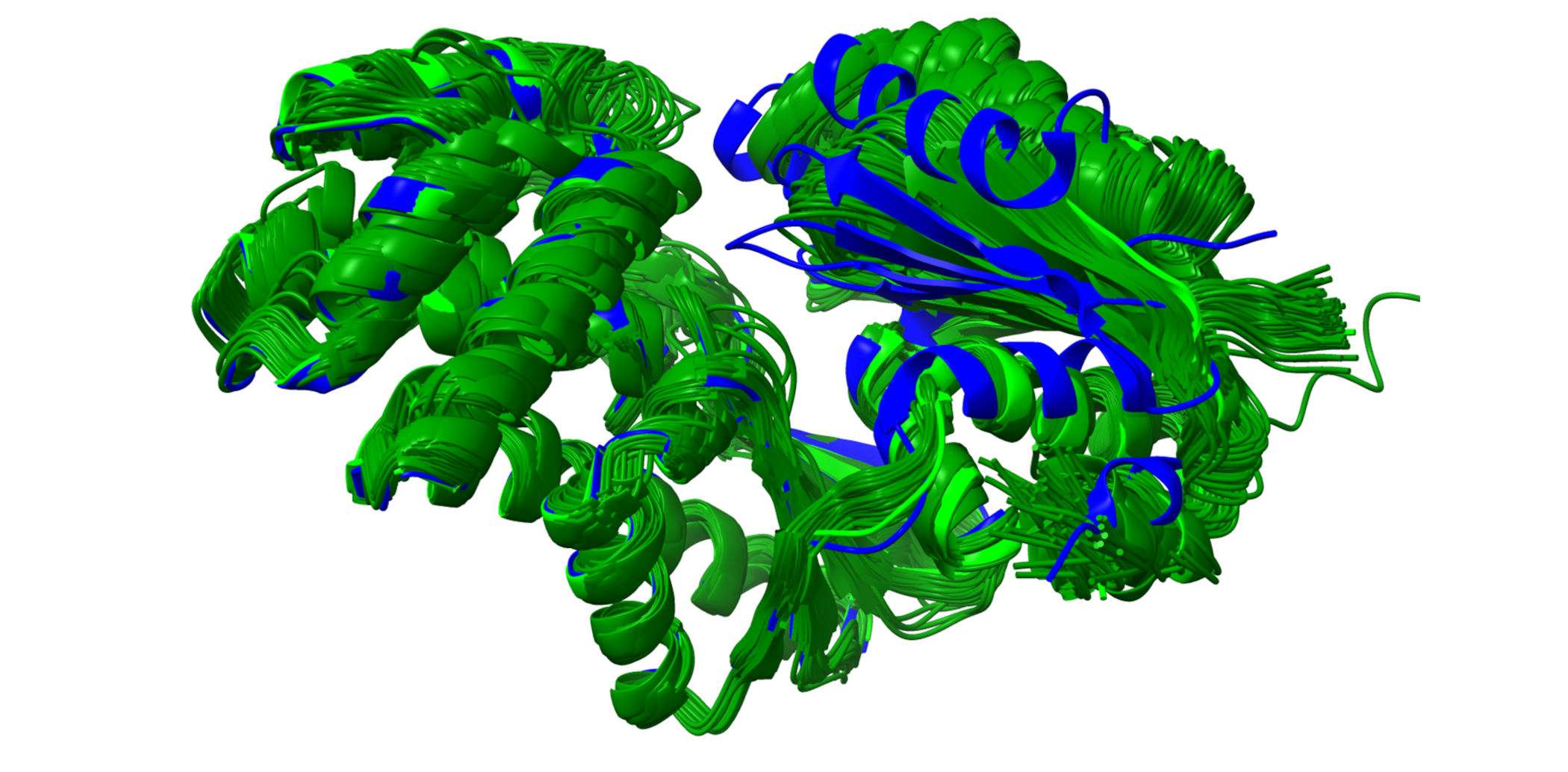

Supplement: Supplementary file 1 [file ijms-25-12968-s001.zip › SUPPLEMENTARY_MATERIALS/FigureS8.tif]

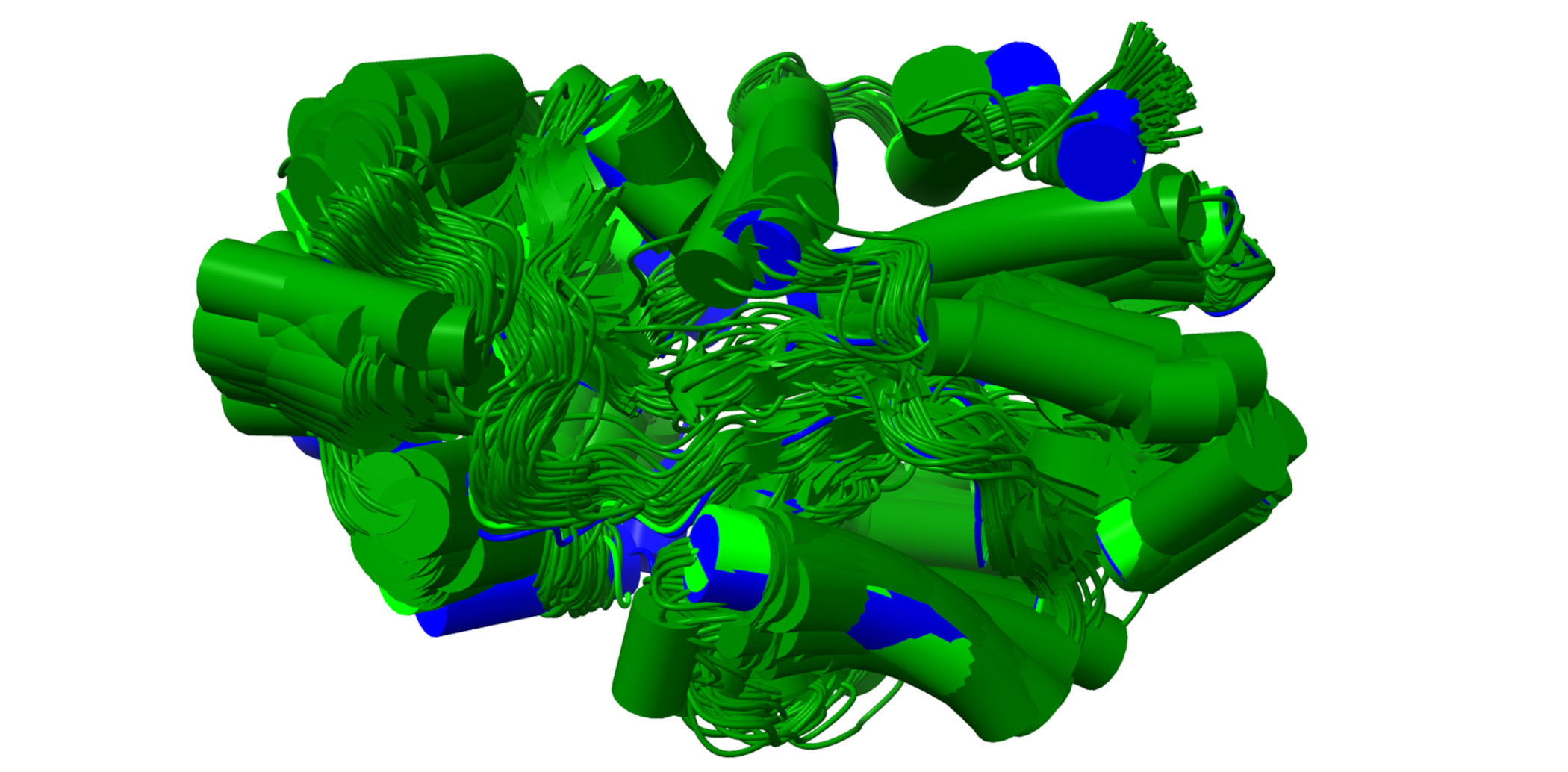

Supplement: Supplementary file 1 [file ijms-25-12968-s001.zip › SUPPLEMENTARY_MATERIALS/FigureS9.tif]
